# Thyrotoxicosis and pregnancy

**DOI:** 10.1186/1756-6614-6-S2-A18

**Published:** 2013-04-05

**Authors:** Małgorzata Gietka-Czernel

**Affiliations:** 1 Department of Endocrinology, Medical Centre of Postgraduate Education, Warsaw, Poland

## 

Thyrotoxicosis affects 1-3.5% of all pregnancies and its major cause is gestational hyperthyroidism and Graves’ disease, less common – multinodular toxic goiter and toxic adenoma, and rare – subacute or silent thyroiditis and struma ovarii.

Gestational hyperthyroidism is a transient form of thyrotoxicosis caused by excessive stimulation of thyroid gland by hCG and usually limited to the first 12-16 weeks of pregnancy. It affects 1-3% of all pregnancies, especially the women with hyperemesis gravidarum and multiple gestation. It can also occur in hydatidiform mole and choriocarcinoma. The rare cause of gestational hyperthyroidism is a functional mutation of TSH receptor which results in hypersensitivity of thyroid gland to hCG.

Graves’ disease is recognized in 0.1-0.4% of all pregnancies. The symptoms of hyperthyroidism usually exacerbate in the first trimester, diminish in the second one and completely subside in the third in 20-30% of affected women. Graves’ disease can be differentiated from gestational thyrotoxicosis by the clinical and laboratory presence of autoimmunity: orbitopathy, typical goiter and TSH receptor antibodies (TRAb).

Multinodular toxic goiter and toxic adenoma are the very rare cases of hyperthyroidism in pregnancy because these conditions typically occur in the older population.

Overt hyperthyroidism constitutes a special risk to mother and fetus. It can precipitate hypertension, congestive heart failure and preeclampsia in pregnant woman. The higher rate of miscarriage, preterm delivery and placental abruption are also noted. In Graves’ disease transplacental passage of TRAb can induce fetal and neonatal hyperthyroidism. The fetal effects can be seen after 20 weeks’ gestation and include tachycardia, cardiomegaly, hydrops, advanced bone age, intrauterine growth restriction and stillbirths. Suppression of the fetal TSH can be the cause of central hypothyroidism later on. Antithyroid drugs (ATDs) also freely cross the placenta and can cause fetal hypothyroidism with bradycardia, growth retardation and delayed bone maturation.

Overt hyperthyroidism due to Graves’ disease, multinodular goiter or toxic adenoma should be treated with ATDs. The aim of ATD therapy is to maintain free HT in the upper limit of the normal reference range or slightly above. The recommended medication in the first trimester is propylthiouracil (PTU) because its use is not associated with congenital abnormalities. Alternatively methimazole (MMI) can be instituted in the case of PTU allergy. In the second and third trimester the use of MMI is preferred because of the possible PTU hepatotoxicity. Serum TSH, and free TH should be monitored every 2-6 weeks. Surgery is indicated in second trimester in the case of severe adverse reactions to ATDs, when high doses of ATDs are persistently needful or when patient is not compliant with ATD therapy. Radioiodine therapy is forbidden in pregnancy because of deleterious effect of radiation on the fetus and possibility of ablation of fetal thyroid when used after 12 weeks’ gestation.

Fetal ultrasound monitoring is recommended in pregnant women with elevated TRAb or treated with ATD from 18-22 or 20-24 weeks’ gestation. The aim is to diagnose fetal thyroid dysfunction. Cordocentesis in an attempt to determine fetal TSH and TH is warranted only in selected cases when clinical data are confounding.

